# Micropropagation of *Agave salmiana*: Means to Production of Antioxidant and Bioactive Principles

**DOI:** 10.3389/fpls.2015.01026

**Published:** 2015-11-23

**Authors:** César A. Puente-Garza, Antonia Gutiérrez-Mora, Silverio García-Lara

**Affiliations:** ^1^Plant-Food Molecular Breeding Unit, Escuela de Ingenieria y Ciencias, Tecnologico de Monterrey, Campus MonterreyMonterrey, Mexico; ^2^Unidad de Biotecnología Vegetal, Centro de Investigación y Asistencia en Tecnología y Diseño del Estado de JaliscoGuadalajara, Mexico

**Keywords:** *Agave salmiana*, micropropagation, axillary shoots, antioxidant activity, nutraceutic

## Abstract

Maguey, *Agave salmiana*, is an important plant for the “pulque” beverage and functional food industries; however, it has several constraints for elite and homogeneous plant production. In this study, a micropropagation process was established to generate *in vitro* plants. The effect of the method on metabolite content and antioxidant (AOX) activity in regenerated plants was evaluated. Young germinated plantlets were micropropagated from axillary shoots using Murashige and Skoog medium supplemented with L2 vitamins, 0.04 mg/L 2,4-dichlorophenoxyacetic acid and 10 mg/L 6-benzylaminopurine. Total soluble sugars from the aqueous fraction and total phenolic acids, total saponins, and AOX activity of the methanol fraction were determined in wild-type (WT) plants, in *in vitro* (IN) plants, and *ex vitro* acclimated plants (EN). The results showed that IN plants have a 50% lower soluble sugar content compared to WT, and EN. The total phenolic acids content was at least 30% higher in micropropagated (IN) and regenerated (EN) plants compared to WT. The total saponin content in IN, and EN plants was 36 and 25 times higher compared to WT. The AOX capacity of IN plants was on average three times higher compared to other treatments. However, no correlation was found between the AOX activity and total phenolic acids or total saponins. A negative and significant correlation (*r* = –0.927; *p* = 0.003) was found between the AOX activity and the total soluble sugars content. Micropropagated plants of *A. salmiana* have a different phytochemical content and bioactivity after the *in vitro* process compared to WT plants. The micropropagation process could be used as a platform for phytochemical enhancement of *Agave* plants.

## Introduction

Agaves are succulent plants native to Mexico, the southwest region of the USA, Central America, and the Canary Islands. Approximately 75% of the species can be found in Mexico, but 74% of these are endemic (Martínez-Salvador et al., 2005). The *Agave* species that produce major revenue in Mexico belong to magueys “pulque". This group is represented by the *Agave americana, A. atrovirens, A. mapisaga*, and *A. salmiana* species (Ortiz-Basurto et al., 2008). The production of these plants in Mexico accounts for 3,681 ha with a possible potential commercial value of USD $100 million.

*Agave salmiana* plants have sexual and asexual reproduction strategies (Arizaga and Ezcurra, 2002). Maturity for sexual reproduction occurs at 10 years of age (Narváez-Zapata and Sánchez-Teyer, 2009), and asexual reproduction occurs in plants at 5 years of age. Rhizome propagation is the most common method for establishing commercial agave plantations (Arizaga and Ezcurra, 2002). However, the lack of sexual reproduction and the generation of clones by rhizome propagation result in very poor genetic variability in agave populations, and genetic improvement of the plant is a very difficult problem to solve (Flores-Benítez et al., 2007). The use of *in vitro* micropropagation has several advantages, such as obtaining populations with elite characteristics, stress tolerance, freedom from pathogens, and stable genetic backgrounds (Domínguez-Rosales et al., 2008). This technique also can provide material for crop improvement using molecular breeding tools (Flores-Benítez et al., 2007). Several studies of *A. salmiana* micropropagation that emphasize the economic and agricultural sustainability and preservation of its diversity have been published (Silos-Espino et al., 2007; Ramírez-Malagón et al., 2008)

Furthermore, agave nutritional properties have been widely documented (Silos-Espino et al., 2007). For example, carbohydrates are one of the most important metabolites for the *Agave* agroindustry (Mancilla-Margalli and López, 2006; Michel-Cuello et al., 2008; Arrizon et al., 2010) due to high accumulation of carbohydrates in the form of fructans, which are mainly composed of sucrose, fructose, and glucose (Mancilla-Margalli and López, 2006). From a nutraceutical point of view, recent reports have highlighted that *Agave* and its by-products have bioactivity due to the content of different metabolites, such as “aguamiel”, the sap of the plant, which contains fructans and saponins (Santos-Zea et al., 2012; Leal-Díaz et al., 2015). The phytochemicals found in *Agave* include phenolic compounds, such as kaempferol and quercetin, in various glycosylated forms (Almaráz-Abarca et al., 2013; Barriada-Bernal et al., 2014), and saponins, as glycosides of hecogenin, diosgenin, chlorogenin, kammogenin, and gentrogenin (Yokosuka and Mimaki, 2007; Pérez et al., 2014). Previous studies have reported an increase in bioactivity of diverse micropropagated plants compared to wild-type (WT) plants (García-Pérez et al., 2012; Dakah et al., 2014), suggesting that the micropropagation of *A. salmiana* could result in this advantage.

Therefore, in this study, a micropropagation process was established to generate *in vitro* plants of maguey *A. salmiana*, and evaluate the effect of this process in terms of total soluble sugars, total phenolic acids, total saponins and antioxidant (AOX) activity in comparison to plants grown from wild populations.

## Materials and Methods

### Plant Material

*Agave salmiana* seeds and plants were provided by the Agmel SA de CV Company (Monterrey, NL, Mexico). The plants and seeds were collected in October 2012, after the rainy season in a commercial plantation. Seeds were taken from dehiscent fruits and germinated in the first 6 months after collection. Selected plants were pathogen and disease-free, and accomplished the standards established for commercial purpose. Two specimens were collected from a 1-year-old plant from a natural population located in the field at Ejido Puebla, Saltillo, Coahuila, Mexico (25°24′54″N; 101°18′11″O; 1442 meters above sea level); the average relative humidity (RH) was 57%, with a precipitation of 16.26 mm during the month. The average temperature registered was 20.5°C, with maximum of 28.8°C and minimum of 10.8°C. For further analysis, the third leaf of each plant was taken as control tissue from WT plants.

### *In Vitro* Seed Germination

In order to establish an optimal germination protocol for further micropropagation the *A. salmiana* seeds were germinated. The seeds were surface-disinfected by soaking them in distilled water containing 1.5% (v/v) of commercial liquid soap and 200 μL of Tween 20^®^ (Sigma–Aldrich, St. Louis, MO, USA), for 2 min. Then they were washed with distilled water for 5 min and placed in a solution of 50% (v/v) commercial bleach (Cloralex^®^, 5.25% w/w, Monterrey, NL, Mexico) for 15 min. The seeds were then submerged in 96% ethanol for 2 min and washed with distilled water. For the germination test, the seeds were divided into four treatment groups: no scarification, chemical scarification, mechanical scarification, and a combination of mechanical and chemical scarification, to determine the optimal method according to the International Seed Testing Association (International seed testing association, 2004). Chemical scarification was conducted by submerging the seeds in H_2_SO_4_ for 1 sec and rinsing immediately with sterile water. Mechanical scarification entailed using a knife to cut the area close to the seed micropyle. Then, groups of 100 seeds were cultivated in jars with 20 mL of freshly prepared Murashige and Skoog (MS) 1:10 w/w (Bairu et al., 2009) solid culture medium at a density of five seeds per jar. The cultures were transferred to an environmental chamber, (Sheldon Manufacturing, Inc., Cornelius, OR) set to 27°C with a photoperiod of 12:12 (12 h of light at 6600 lux and 12 h of dark). Light intensity was set up to 6600 lux (Light meter Model 3251 Traceable^®^, Control Company, Friendswood, TX, USA). The optimal treatment with a maximal number of seeds germinated was selected for further micropropagation processing.

### Optimal *In Vitro* Growth Conditions for Multiplication

Three-week-old plantlets obtained from the *in vitro* germinated seed were multiplied using the axillary shoot method (Santacruz-Ruvalcaba et al., 1999). After removing the roots, one single plant per jar was cultured with 20 mL of solid freshly prepared MS culture medium modified with L2 vitamins (MS + L2) (Santacruz-Ruvalcaba et al., 1999). After 2 weeks, combinations of 6-benzylaminopurine (BAP; Sigma–Aldrich, St Louis, MO, USA; 0.5, 1.0, 5.0, and 10.0 mg/L) and 2,4-dichlorophenoxyacetic acid (2,4-D; Sigma–Aldrich, St Louis, MO, USA; 0.01, 0.025, and 0.04 mg/L) were added to new MS + L2 solid culture medium to identify the optimal concentration for growth, and the highest multiplication rate (Santacruz-Ruvalcaba et al., 1999). All culture media contained 30 g/L sucrose (Sigma–Aldrich) and 4 g/L Phytagel^®^ (Sigma–Aldrich). The pH was adjusted to 5.8 and plant growth regulators were added before autoclaving (20 min at 1 atm and 121°C). The cultures were transferred to a chamber with the temperature set to 27°C and a photoperiod of 16:8 h light:dark (6600 lux). Axillary shoots and the presence of a callus were quantified after 60 days (Santacruz-Ruvalcaba et al., 1999). The optimal plant growth regulator concentration allowing the maximum number of axillary shoots was selected for further multiplication. Once the plantlets were multiplied, they were placed in acclimatization medium free of plant growth regulators for 30 days for root growth.

### Acclimatization

Rooted plants were removed from the culture medium and placed in trays with a wet soil mixture of 7:3 (v/v) peat moss and vermiculite (Cosmopeat and Cosmocel; Monterrey, NL, Mexico); the soil was mixed with a granular fertilizer (Osmocote Classic, Scotts, CA Geldermalsen). The plants were subsequently transferred and maintained at 34°C and 95% RH for 1 week, after which the RH was adjusted manually to 34% for another week. After acclimatization, plants produced *in vitro* were grown at the nursery facilities of Tecnológico de Monterrey, Monterrey, NL, Mexico (25°38′43. 93″N; 100°17′01. 07″O; 532 meters above sea level) using standard agronomic practices for WT succulent plants. Plants were exposed to open environment conditions during March of 2013, with a RH 49.2%, 15.5 mm of precipitation, and an average temperature of 20.4°C, with maximum of 28.9°C, and minimum of 14.4°C. The survival rate of the plants was calculated after 1 month.

### Preparation of Plant Samples

Leaf tissue samples were taken from the *in vitro* (IN) plants obtained from the multiplication step, *ex vitro* acclimated plants obtained from open environment conditions (EN), and WT plants obtained from a natural population, recollected after rainy season. All samples were stored at –80°C overnight and then lyophilized. The dried leaf tissue was ground using a mixer ball mill (MM 400; Retsch/Verder Scientific, Col. Germany) and stored at –20°C for analysis.

### Quantification of Total Soluble Sugars

For the extraction of soluble sugar dried leaf tissue (100 mg) was homogenized using 1 mL of distilled water. The samples were placed in a shaking incubator (VorTemp 1550, Labnet Int. Inc., Edison, NJ, USA) for 7 h at 70°C and 150 rpm. The extracts were centrifuged at 5000 rpm for 5 min and the supernatant was vacuum dried and re-suspended in 1 mL of water (Arrizon et al., 2010). Reducing sugars were quantified following the DNS protocol modified by King et al. (2009). Briefly, in a 96-well microplate, 60 μL of samples and standards (glucose) were placed in wells in triplicate. Then, 120 μL of DNS reagent was added. The microplate was placed in a shaking incubator (VorTemp 1550, Labnet Int. Inc., Edison, NJ) for 15 min at 95°C and 150 rpm. The reaction was stopped by placing the microplate at 4°C for 5 min and the absorbance was measured at 540 nm. The results were expressed as mg of dextrose equivalents (DE) per g of dry weight (dw).

### Phenolic Acids and Saponins Extraction

One hundred milligram of dried leaf tissue was homogenized for the extraction using 1 mL of a methanol–water 80:20 (v/v) solution. The extract was placed in a shaking incubator (VorTemp 1550; Labnet Int. Inc., Edison, NJ, USA) for 2 h at 150 rpm and 30°C; then, it was centrifuged at 3000 rpm for 5 min. The supernatant was vacuum dried, suspended in 1 mL methanol–water 50:50 (v/v), and used for the analysis of total phenolic acids, total saponins, and AOX activity (García-Pérez et al., 2012).

### Total Phenolic Acids Quantification

Total phenolic acids concentration was determined using Folin–Ciocalteu reagent and gallic acid as a standard according to the method of García-Pérez et al. (2012). Samples (20 μL) were introduced into a microplate, after which 100 μL of 10% of the Folin–Ciocalteu reagent and 80 μL of Na_2_CO_3_ 7.5% (w/v) were added. After incubating for 1.5 h at 30°C, the absorbance was measured at 765 nm using a microplate reader (Synergy^TM^ HT Multi-Detection; BioTek Inc., Winooski, VT, USA). The results were expressed as mg of gallic acid equivalents (GAE) per g of dw.

### Total Saponins Quantification

Total saponins were quantified as protodioscin equivalents (PE; Sigma–Aldrich, St. Louis, MO, USA) as detected by HPLC-ELSD (Agilent Technologies, 1200 series, Santa Clara, CA, USA, evaporative light scattering detector) with a Zorbax Eclipse XDB-C18, 4.6 mm × 150 mm (5 μm) column (Agilent Technologies, Santa Clara, CA, USA) using nitrogen as the drying gas, pressure at 3.8 bar and tube temperature of 45°C, as previously reported (Leal-Díaz et al., 2015). A gradient elution proposed by Leal-Díaz et al. (2015) was improved to enhance the resolution of saponins. Briefly, the program consisted of phase A (water and 0.1% formic acid) and phase B (acetonitrile with 0.1% formic acid) at a flow rate of 0.8 mL/min. The gradient was as follows: 82% of phase A was maintained during the first 15 min; decreased to 25% over 10 min; maintained for 5 min before reducing to 0% over 10 min; and maintained at 100% B for the last 10 min. The data were collected and analyzed by “Chem Station for LC 3D systems” (Agilent Technologies^®^, Santa Clara, CA, USA), provided with the equipment. The standard curve of protodioscin was constructed from 10 to 500 ppm and the content was determined using the area under the curve of peaks.

### Antioxidant Activity of Plant Extracts

Antioxidant activity was determined using the oxygen radical absorbance capacity assay. Extracts were evaluated following the method described by García-Pérez et al. (2012), using a standard of trolox (Sigma–Aldrich) with fluorescein (Sigma–Aldrich). Peroxyl radicals were generated by adding 2,2′-azobis (2-amidinopropane) dihydrochloride (Sigma–Aldrich), and the fluorescence loss signal was monitored in a microplate reader. The absorbance of excitation and emission were set at 485 and 538 nm, respectively. The results were expressed as μmol of trolox equivalents (TE) per g dw.

### Statistical Analysis

For the micropropagation protocol, a 4 × 5 factorial design was used to obtain the maximal number of shoots. Germination, total shoots, total soluble sugars content, total phenolic acids content, total saponins content, and AOX activity capacity analysis were subjected to analysis of variance and Pearson correlation analysis using the statistical software Minitab 16. Differences among means were compared with Tukey’s tests at *p* < 0.05. Correlation analyses were performed for the primary phytochemical compounds bioactivity at different levels of *p*.

## Results

### Optimal Germination and Growth Conditions

*Agave salmiana* seeds were germinated *in vitro*, establishing an optimal protocol for further micropropagation. Four seed treatments were evaluated. A combination of mechanical and chemical scarification had a germination rate of 93% after 11 days of imbibition. However, no significant difference was found in comparison with the seeds treated with mechanical scarification, which had a 90% germination rate after 4 days of imbibition. No scarification had a rate of 3%, while only chemical scarification had the lowest rate of germination (0%).

### *In Vitro* Multiplication and Micropropagation Process

**FIGURE 1:**
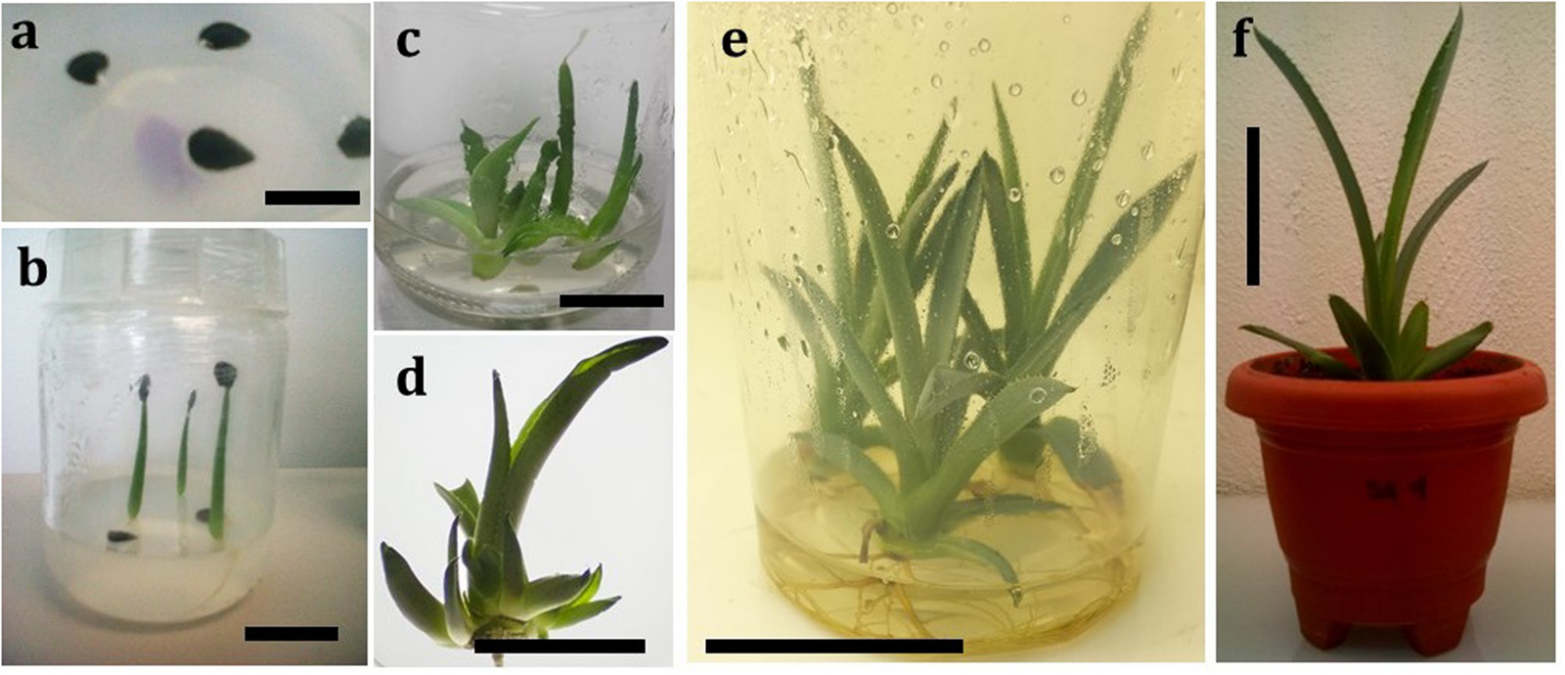
**Micropropagation steps in *Agave salmiana*.** **(a)** Seeds germinated *in vitro*. The seeds were taken from a mature plant bearing an inflorescence, bar = 1 cm; **(b)** One-week old plantlet from seed, bar = 2.5 cm; **(c)** Initial shoot-tip culture after germination, bar = 1 cm; **(d)** Axillary shoots induction response of explants, bar = 5 cm; **(e)** Multiplication and rooting of axillary buds, bar = 5 cm; **(f)** Acclimatization, bar = 5 cm.

After seed germination of *A. salmiana*, an improved micropropagation protocol was established to regenerate plants using both BAP and 2,4-D as plant growth regulators. **Table 1** summarizes the results of axillary shoot generation. The optimal combination of these plant growth regulators to produce the highest number of axillary shoots occurred when a ratio of 250 parts BAP to 1 part 2,4-D was maintained In the absence of 2,4-D, an increase in the concentration of BAP had no significant effect on the offshoot generation. At the highest concentration of 2,4-D, the generation of axillary shoots was totally inhibited. The proposed protocol allows the regeneration of whole plants from WT genotypes in 16 weeks using germinated young plantlets (**Figure 1**). In the first stage, the germinated seeds were established (**Figure 1a**), and after 2 weeks, when the plantlets reached 2–3 cm (**Figure 1b**), they were transferred to MS + L2 medium after removing the roots (**Figure 1c**). After 60 days in MS + L2 medium and plant growth regulators, the plantlets generated axillary shoots that could easily be identified and separated easily for further multiplication (**Figure 1d**). Spontaneous rooting was observed in 100% of axillary buds established in plant growth regulator-free MS + L2 medium after 30 days (**Figure 1e**). Acclimatization of the rooting plants was achieved after 2 weeks (**Figure 1f**), with a 90% survival rate of the transferred plants.

**Table 1:** Number of axillary shoots generated after 60 days using a combination of 2,4-D and BAP under *in vitro* conditions by the axillary shoots generation technique.

BAP (mg/L)	2,4-D (mg/L)			
	
	0.00	0.01	0.02	0.04
0.0	0.66 ± 0.57 fgˆ*	0.25 ± 0.50 fg	0.00 ± 0.00 g	1.66 ± 1.52 fg
0.5	3.00 ± 1.00 defg	2.75 ± 0.95 efg	0.00 ± 0.00 g	0.00 ± 0.00 g
1.0	1.33 ± 0.57 fg	2.66 ± 1.15 efg	2.33 ± 1.15 efg	0.33 ± 0.57 fg
5.0	2.33 ± 0.57 efg	6.00 ± 2.10 cde	10.33 ± 0.57 ab	0.00 ± 0.00 g
10.0	3.50 ± 2.74 ef	2.00 ± 1.41 efg	7.33 ± 1.15 bcd	14.00 ± 0.70 a


*^∗^Different letter(s) denote statistically significant differences at *p* < 0.05. BAP, 6-benzylaminopurine. 2,4-D, 2,4-dichlorophenoxyacetic acid.*

**FIGURE 2:**
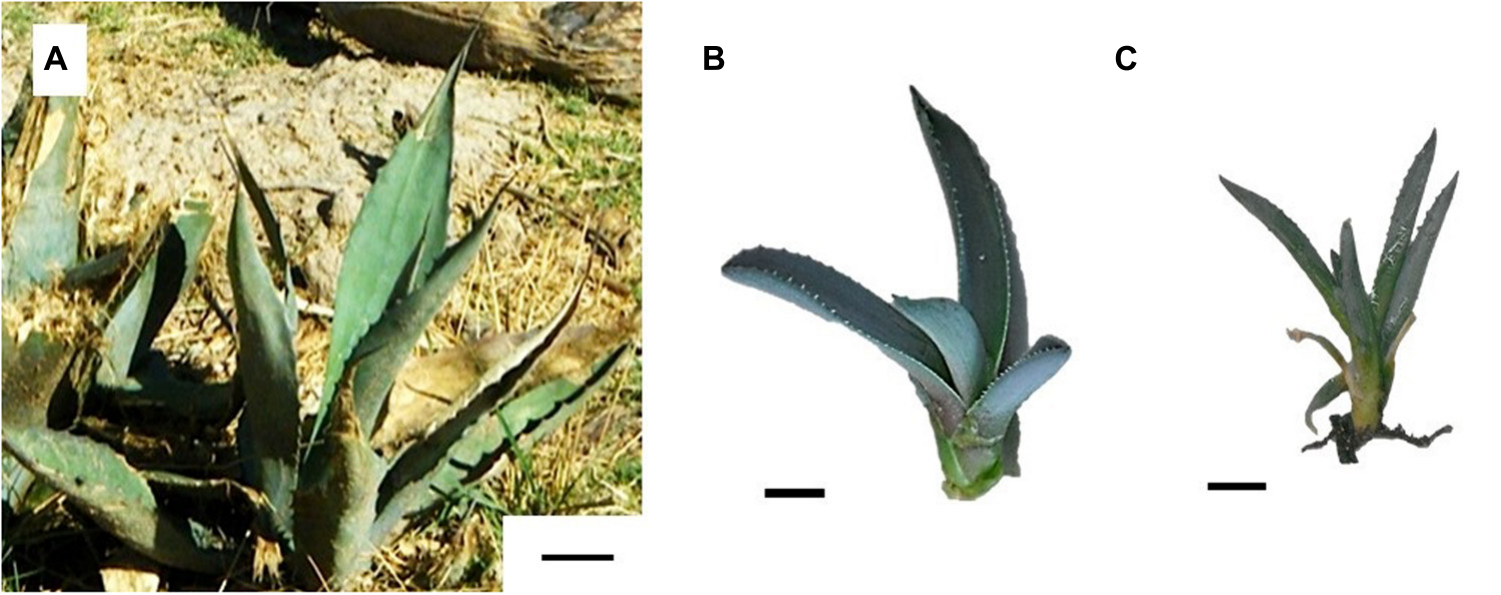
**Comparison of *Agave salmiana* plants analyzed.** **(A)** Wild-type plant (WT, bar = 5 cm); **(B)** *In vitro* generated plant (IN, bar = 1 cm); **(C)** *Ex vitro* acclimated (EN, bar = 1 cm).

**FIGURE 3:**
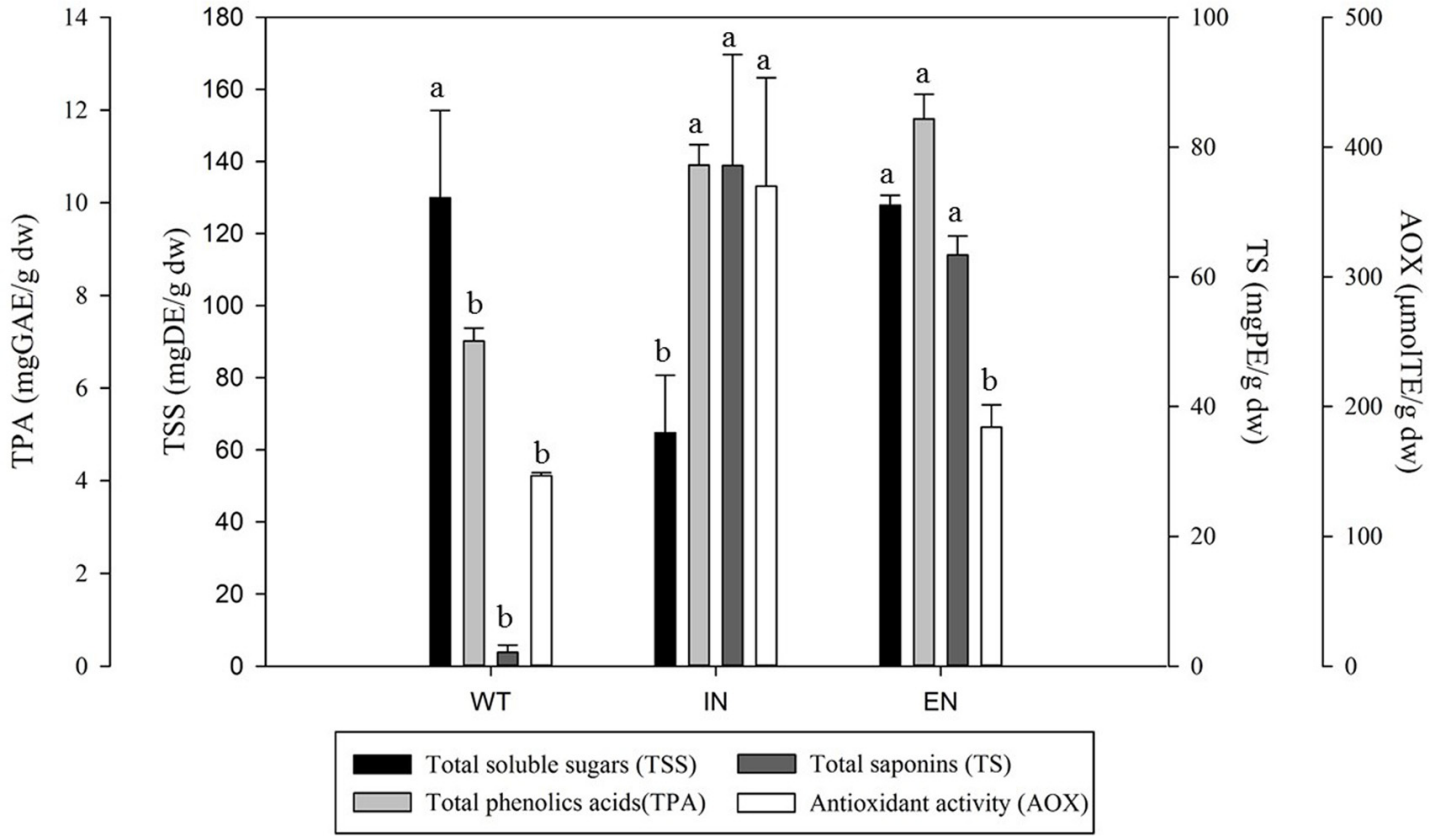
**Total soluble sugars, phenolic acids, saponin content and antioxidant activity of *Agave salmiana* extracts from WT, an *in vitro* environment (IN), and *ex vitro* acclimated plant (EN).** Values expressed on a dry basis.

With this micropropagation process, plant tissues were collected from three major steps: (1) *in vitro* (IN, **Figure 2B**) plants obtained from the tissue culture laboratory at the multiplication step, (2) *ex vitro* plants obtained after acclimatization step (EN, **Figure 2C**), and (3) WT plants (WT, **Figure 2A**) obtained from a natural population.

### Quantification of Sugars, Phytochemicals, and Antioxidant Activity

An aqueous plant tissue extract was used to evaluate the content of total soluble sugars (**Figure 3**). A significant difference (*P* < *0.05*) in the content of soluble sugars was observed between the micropropagation process steps. Two groups of response were identified: WT and EN (129 and 127 mg DE/g dw, respectively) and IN (64 mg DE/g dw) with a significant difference. Compared with WT and EN, the IN plants contained 50% less of the total reducing sugars.

The total phenolic acids content in WT, IN, and EN *A. salmiana* plants is presented in **Figure 3**. There were significant differences (*P* < 0.05) between the micropropagated and regenerated plants, compared with WT plants. The range of total phenolic acids content varied between 7 to 11 mg GAE/g dw. The IN and EN plants had the highest phenolic acids content (11.8 and 10.8 mg GAE/g dw). The WT plants showed the lowest phenolic acids content, with 7.0 mg GAE/g dw. Compared with WT, there was an increase of 35 and 40% in the phenolic acids content in the IN, and EN plants, respectively.

Total saponins content was quantified from the methanolic extracts, and the results are presented in **Figure 3**. The saponin content showed significant differences (*P* < 0.05) between plants resulting from the micropropagation process. The lowest saponin content was observed in WT plants (2.1 mg PE/g dw). In contrast, IN and EN plants had the highest total saponin content (77.1, 63.3 mg PE/g dw, respectively). Micropropagated and regenerated plants (IN and EN) showed an increase of 36 and 29 times in saponin contents compared with the WT plants.

Quantification of the AOX activity was performed using the methanolic extract where total phenolic acids and saponins were quantified (**Figure 3**). The AOX activity was significantly different among plants from different micropropagation steps (*P* < 0.05). Plants from IN showed the highest AOX activity (369 μmol TE/g dw), followed by EN and WT, (184 and 146 μmol TE/g dw, respectively). Direct comparisons of IN with EN and WT showed that AOX activity was 2.5-fold and 2.8-fold higher, respectively.

Pearson correlation analysis showed a lack of correlation between AOX activity with total phenolic acids and total saponins (*r* = 0.327, *p* = 0.475; *r* = 0.600, *p* = 0.154, respectively). A negative relation was found between AOX activity and the total soluble sugar content (*r* = –0.927, *p* = 0.003).

## Discussion

### *In Vitro* Seed Germination

Poor rates of seed germination have been observed in agave fields in the north of Mexico (Vázquez-Díaz et al., 2011). Although agave seeds have low availability and low viability, Ramírez-Tobías et al. (2011) determined that seedlings are 50% more vigorous than those obtained by offshoots and therefore seed generated plants have advantages. This study presents an alternative germination protocol to that proposed by Ramírez-Tobías et al. (2011), although they achieved a germination rate of 90% after 3 days of imbibition at 25°C, it was necessary to address the poorer seed quality of seed obtained from plants in the north of Mexico in order to generate enough plantlets for the micropropagation process. Seeds of *A. salmiana* collected in Nuevo Leon, Mexico for this study also presented low levels of germination (3%). In this study the use of mechanical scarification was optimal to produce a high number of plantlets with a 90% germination after 4 days, comparable with the results of Ramírez-Tobías et al. (2011) without treatment. Variations in the rate of germination have been attributed to the lack of seed dormancy (Ramírez-Tobías et al., 2011), or related to the use of sand versus culture media. The need for seed treatment revealed in this study has also been associated with geographic origin, genetic variance, and storage conditions.

### Optimal *In Vitro* Multiplication

In the Agavaceae family, several improved methods for plant micropropagation have been reported. Compared with those studies, the micropropagation process presented in this study for *A. salmiana* is more efficient in terms of time for regeneration compared with the study by Ramírez-Malagón et al. (2008) for *A. tequilana* and Santacruz-Ruvalcaba et al. (1999) for *A. parrasana* Berger.

The use of 2,4-D and BAP is common for generating axillary shoots in different species, such as *A. parrasana* Berger (Santacruz-Ruvalcaba et al., 1999) and *A. tequilana* (Ramírez-Malagón et al., 2008). However, previous studies have also reported controversial results regarding the use of auxins in the production of axillary buds in the Agavaceae family: e.g., *A. parrasana* by Santacruz-Ruvalcaba et al. (1999), and *A. Americana* by Chen et al. (2014). In this study, a combination of 2,4-D and BAP generated a high number of axillary shoots without an observed negative effect.

Compared with other strategies of micropropagation for *A. salmiana* axillary shoots, the method was 79% more efficient in the plants produced than reported by Ramírez-Malagón et al. (2008) for the same time period. Micropropagation comparisons reveal differences in terms of the type of explant used. For example, using cambium meristem or cylindrical core in *A. salmiana*, Silos-Espino et al. (2007) obtained a yield of 20 axillary shoots in 45 days, which is 30% more efficient than the current study. These differences are attributed to the seed-plantlets used in this study as the main explant, as observed by Zhang et al. (2013), where the explant and variety used had a direct impact on the shoot efficiency.

Surprisingly, the spontaneous rooting observed in this study is contrary to the results reported by Silos-Espino et al. (2007) for *A. salmiana*, in which 0.2 mg/L of IAA was necessary to generate a complete root system in 45 days. This response is common in *Agave* species and confirmed that *A. salmiana* does not require auxins to produce roots, similar to *A. parrasana* Berger (Santacruz-Ruvalcaba et al., 1999) and *A. hybrid* No. 11648 (Zhang et al., 2013). Additionally, the spontaneous rooting might be due the absence of BAP and its derivative because the presence of cytokinins inhibits the formation of roots and correct development of plants during the acclimatization process (Aremu et al., 2015). Most *Agave* species have high survival rates (80–98%) after acclimatization (Silos-Espino et al., 2007; Zhang et al., 2013; Chen et al., 2014). Based on these results, this study supports the massive use of seeds in the micropropagation of *A. salmiana*, which has been previously demonstrated to ensure large numbers of specimens, genetic variability generation, diversity, sanitation, and stable genetics (Portillo et al., 2007).

### Sugar and Phytochemicals in the Micropropagation Process

Soluble carbohydrates such as fructose and glucose are the main source of energy in *Agave* sp. and are obtained by the hydrolysis of sucrose by the enzyme invertase (Michel-Cuello et al., 2008). Total soluble sugars in WT and *ex vitro* plants of *A. salmiana* account for 12% of the total weight, which is similar to the 15% of free sugars observed in 2-year-old plantlets of *A. tequilana* (Arrizon et al., 2010). Previous studies have shown that the amount of total carbohydrates in *Agave* sp. increases with the age of the plant (Pinos-Rodríguez et al., 2008) and that these carbohydrates are commonly accumulated as fructans (Arrizon et al., 2010). However, no significant difference in the amount of soluble sugars extracted as fructose and glucose has been reported because these sugars are used by the plant immediately, either for the formation of fructans or for synthesis of sucrose as a transport sugar (Michel-Cuello et al., 2008).

A study by Michel-Cuello et al. (2008) found no significant difference between total soluble sugars and different ages of *A. salmiana* plants (immature and mature). However, in this study significant differences were observed between WT, *in vitro* and *ex vitro* plants. In fact, the low content of total soluble sugars obtained in the *in vitro* plants is consistent with the report of Barreto et al. (2010), where a decrease of 90% in the content of fructose was observed once the plants are in an *in vitro* environment. This reduction is explained by the addition of exogenous sucrose and the limited light intensity of the *in vitro* culture. These conditions have an effect on the net photosynthesis rate because glucose, a reducing and soluble sugar, is synthesized only during photosynthesis in leaves (Michel-Cuello et al., 2008).

WT plants of *A. salmiana* exhibited a similar range of total phenolic acids content compared with *A. americana* (between 8 and 12 mg/g dw of leaf) in two previous studies (Hamissa et al., 2012; Nasri and Ben Salem, 2012). Nevertheless, an increase in total phenols was observed when plants were cultivated *in vitro*, and total phenolics decreased when the plants were cultivated *ex vitro*. Those changes are consistent with the role that phenolic compounds play in response to abiotic stress such as drought (Almaráz-Abarca et al., 2013). The accumulation of phenolic acids has been related to an increase in activity of phenylpropanoid pathway enzymes, such as CHS and PAL (Yuan et al., 2012), due to oxidative stress caused by the absence of an abundance of water (Shan and Liang, 2010).

The total saponin content in IN and EN plants was 7- and 6-times higher in comparison to that reported by Pinos-Rodríguez et al. (2008) for 16-year-old plants of *A. salmiana* (11.1 mg/g dw); however, WT plants from the north of Mexico had a 5-times lower saponin content. The content of saponins is higher in young plants compared with mature or old plants (Francis et al., 2002). In comparison with other species, all steps of micropropagation (IN and EN) were 13 and 11-times higher than reported for *A. duranguensis* (5.7 mg/g dw; González-Valdez et al., 2013), and 7- and 6-times higher than reported for *A. lechuguilla* (10.4 mg/g dw; Hernández et al., 2005).

In this study, increases in the total saponins content from *in vitro* to WT in the micropropagation steps are consistent with the changes reported for *in vitro* cultivated *Panax ginseng* compared to a naturally cultivated 4–6-year-old plant (68% more), and this increase was attributed to the effect of growth regulators in the medium (Zhong et al., 1996). Lian et al. (2002) reported a 50% increase of total saponin content in *Panax ginseng* when BAP was added from 0 mg/L to 0.5 mg/L.

### Antioxidant Activity of Plant Extracts

Previous studies have reported AOX activity in *Agave* species. Studies with *A. rzedowskiana* have shown a high AOX capacity of 862 μmol TE/g dw (Ahumada-Santos et al., 2013) and low levels in *A. americana* (70 μmol TE/g dw; Chirinos et al., 2013). However, compared to this study, *A. salmiana* showed intermediate values of AOX activity (369 μmol TE/g dw). In a broader comparison with 42 elite AOX plants, fruits, vegetables (Pertuzatti et al., 2014), and maize (Urias-Peraldí et al., 2013), *A. salmiana* leaves were considered a medium source of AOXs. Upon comparison with other crops, such as wheat (58–270 μmol TE/g dw; Malunga and Beta, 2015) or sorghum (70–204 μmol TE/g dw; Awika et al., 2009), *A. salmiana* presented higher values, which might constitute an alternative source of AOXs.

The increase in AOX activity *in vitro* plants has been reported only in *Ziziphora teniur* L. The mechanism that explains the increase is still unclear, although it is suggested that the presence and interaction of cytokinins and auxins plays an important role in increasing this bioactivity (Dakah et al., 2014). Alternatively, Zhang et al. (2014) proposed that the presence of molecules such as phenols or saponins is involved in the increase in AOX activity. Finally, and after the micropropagation process, plants *ex vitro* return to low levels of AOX activity. This is explained based on previous work that reported after re-watering, limited-irrigated plants return to their normal photosynthetic activity levels (Campos et al., 2014).

Several studies have related the phytochemical content with AOX activity. For example, a positive correlation between total phenolic acids content and AOX has been reported in extracts of desert plants (Hamissa et al., 2012). In contrast, in this study, no relationship between AOX and phenolic acids was found. Furthermore, AOX activity has been associated with non-phenolic acid molecules present in the extract (Ribeiro et al., 2013; Zhang et al., 2014). For example, in *A. sisalana* waste, AOX activity was associated with the presence of tigogenin, homoisoflavonoids, and flavones (Zhang et al., 2014). It is important to mention that recent studies in *A. sisalana* have shown that the combination of phenolic compounds and saponins in the raw extract had a higher AOX activity compared with isolated components (75% less than raw extract; Ribeiro et al., 2013). In this study, total saponins had an insignificant correlation with the bioactivity (*r* = 0.600; *p* = 0.154), and the sum of total saponins and total phenolics acids did not correlate with the bioactivity (*r* = 0.327; *p* = 0.475). Current evidence demonstrates that *A. salmiana* plant extracts are composed of kaempferol and quercetin (Almaráz-Abarca et al., 2013), while saponins consist of hecogenine, diosgenin, and chlorogenin glycosides (Yokosuka and Mimaki, 2007; Leal-Díaz et al., 2015). These findings suggest that the difference in the specific extracted compounds caused a difference in AOX activity. Negative correlation between AOX activity and total soluble sugars (*r* = –0.927; *p* = 0.003), is explained by the changes in the plant growth conditions. *In vitro* environment is a stress factor compared with *ex vitro*. It has been reported a reduction in the accumulation of sugars under abiotic stress as a way to generate mechanisms of defense against abiotic factors (Suárez-González et al., 2014). In contrast, *ex vitro* plant metabolism is directed to the accumulation of carbohydrates (primary metabolism). Further studies are needed to establish which specific molecules of phenolic acid compounds, saponins or the ratio between them, are correlated with the bioactivity observed in *A. salmiana* in the present study. In the near future, this micropropagation process can be used as a platform for the enhancement of specific metabolites and bioactivity in *A. salmiana* plants.

Finally, changes that occur during the micropropagation process in terms of total soluble sugars, phenolic compounds, saponin content and AOX activity, have not been studied in detail. The process of micropropagation in *A. salmiana* generated changes in the AOX activity of the extracts. Changes in the contents of saponins and phenolic compounds in the different plant steps did not correlate with the AOX activity. Further investigation is necessary to determine which specific metabolites are responsible for AOX in the extracts.

## Author Contributions

All authors contributed equally to this work. CP-G, performed all experiments and phytochemical determinations; AG, developed the micropropagation protocol; SG, developed the strategy and experiments design. All authors wrote and reviewed the latest version of this manuscript.

## Conflict of Interest Statement

The authors declare that the research was conducted in the absence of any commercial or financial relationships that could be construed as a potential conflict of interest.
